# Assessment of Stiffness of Large to Small Arteries in Multistage Renal Disease Model: A Numerical Study

**DOI:** 10.3389/fphys.2022.832858

**Published:** 2022-03-30

**Authors:** Hasan Obeid, Vasiliki Bikia, Catherine Fortier, Mathilde Paré, Patrick Segers, Nikos Stergiopulos, Mohsen Agharazii

**Affiliations:** ^1^CHU de Québec Research Center, L’Hôtel-Dieu de Québec Hospital, Québec City, QC, Canada; ^2^Division of Nephrology, Department of Medicine, Faculty of Medicine, Université Laval, Québec City, QC, Canada; ^3^Laboratory of Hemodynamics and Cardiovascular Technology, Swiss Federal Institute of Technology Lausanne, Lausanne, Switzerland; ^4^Department of Médicine, Research Centre of the Hôpital du Sacré-Coeur de Montréal, Université de Montréal, Montréal, QC, Canada; ^5^BioMMeda – Institute for Biomedical Engineering and Technology, Ghent University, Ghent, Belgium

**Keywords:** kidney failure, pulse wave velocity, blood pressure, 1-D modeling, arterial tree

## Abstract

Arterial stiffness (AS), as assessed *via* pulse wave velocity (PWV), is a major biomarker for cardiovascular risk assessment in patients with chronic kidney disease (CKD). However, the mechanisms responsible for the changes in PWV in the presence of kidney disease are not yet fully elucidated. In the present study, we aimed to investigate the direct effects attributable to biomechanical changes in the arterial tree caused by staged renal removal, independent of any biochemical or compensatory effects. Particularly, we simulated arterial pressure and flow using a previously validated one-dimensional (1-D) model of the cardiovascular system with different kidney configurations: two kidneys (2KDN), one single kidney (1KDN), no kidneys (0KDN), and a transplanted kidney (TX) attached to the external iliac artery. We evaluated the respective variations in blood pressure (BP), as well as AS of large-, medium-, and small-sized arteries *via* carotid-femoral PWV (cfPWV), carotid-radial PWV (crPWV), and radial-digital PWV (rdPWV), respectively. Our results showed that BP was increased in 1KDN and 0KDN, and that systolic BP values were restored in the TX configuration. Furthermore, a rise was reported in all PWVs for all tested configurations. The relative difference in stiffness from 2KDN to 0KDN was higher in the case of crPWV (15%) in comparison with the increase observed for cfPWV (11%). In TX, we observed a restoration of the PWVs to values close to 1KDN. Globally, it was demonstrated that alterations of the outflow boundaries to the renal arteries with staged kidney removal led to changes in BP and central and peripheral PWV in line with previously reported clinical data. Our findings suggest that the PWV variations observed in clinical practice with different stages of kidney disease may be partially attributed to biomechanical alterations of the arterial tree and their effect on BP.

## Introduction

Arterial stiffness (AS) is an important mediator in the development of adverse cardiovascular outcomes and has become one of the most important biomarkers to predict risk in patients with chronic kidney disease (CKD), arterial hypertension, and other cardiovascular diseases (Laurent et al., 2001; Vlachopoulos et al., 2010; Chirinos et al., 2019). Through dysregulation of various biological mechanisms, the uremic milieu plays a significant role in the cumulative vascular damage that results in AS which can be evaluated along different arterial pathways or segments (Pannier et al., 2005; Utescu et al., 2013; Fortier et al., 2015). The “Expert consensus document on arterial stiffness” (Laurent et al., 2006) has defined carotid-femoral pulse wave velocity (cfPWV) as the reference technique for the noninvasive measurement of AS due to two major reasons: (i) for being a strong, independent risk factor of morbidity and mortality (Ben-Shlomo et al., 2014) and (ii) for effectively representing the stiffness of the aorta, which constitutes the largest elastic artery in the body and the most significant contributor to the buffering capacity of the systemic circulation (Stergiopulos et al., 1994).

PWV relates to both the intrinsic elasticity of the arterial wall and its anatomic dimensions, as expressed by the Moens–Korteweg and Bramwell–Hill equations (Bramwell and Hill, 1922). Measurement of PWV is simple and straightforward, and it is derived as the ratio of the distance between two measuring arterial locations and the time required for the pulse wave to travel from one location to the other (i.e., the pulse transit time, PTT). In addition to aortic PWV, indices of peripheral AS have also been associated with adverse clinical outcomes in kidney failure patients (Fortier et al., 2015).

Aortic stiffness enhances the transmission of pulsatile energy, an increase pulse pressure (PP), into the microcirculation of vital high-flow low-resistance organs such as kidneys, where it may lead to tissue injury (Chirinos et al., 2019). At the same time, a decline in kidney function results in biological alterations, such as endothelial dysfunction and vascular calcification, leading to increased AS (Bruno et al., 2013, 2016). In addition to these structural and functional alterations of the vasculature, a decline in kidney function also leads to an increased resistance of the renal vasculature. This increase in resistance is accompanied by reduced renal blood flow, which, in itself, might alter the behavior of the vascular tree and increase vascular stiffness.

The renal circulation is characterized by high flow and low resistance, which explains why a shunting of blood away from a defective kidney could significantly alter cardiovascular physiology. More simply put, the “absence” of one or both kidneys could affect AS from a biomechanics stand-point on the cardiovascular system in kidney disease. In order to examine this hypothesis, we used a validated human arterial tree model to assess the impact of an altered renal circulation on AS in model configurations representative of different stages of renal disease. Specifically, our objectives were: first, to adapt a human 1-D arterial tree model to create configurations that would represent the mechanical alterations occurring with staged removal of the kidneys and, second, to use these simulated configurations to assess changes in AS of three arterial regions corresponding to large (cfPWV), medium (carotid-radial PWV, crPWV), and small-sized vessels (radial-digital PWV, rdPWV; Obeid et al., 2021), respectively. Inputting different configurations into the *in silico* model, we ran numerical simulations designed to mimic different degrees of reduced renal function and examined the variations in blood pressure (BP), PWV and flow at different arterial regions. Additionally, PWV values were calculated under different degrees of stiffness of the arterial tree, by modifying the distensibility of vascular segments of interest in both a uniform and non-uniform manner.

## Materials and Methods

We used a human 1-D arterial tree model and adapted it to create configurations representing different stages of renal disease. Subsequently, we ran numerical simulations to determine cfPWV, crPWV, and rdPWV for the various configurations.

### Brief Description of the 1-D Arterial Tree Model

The *in silico* 1-D cardiovascular model adopted in this study has been previously described by Reymond et al. (2009). This model provides physiological, realistic-close pressure and flow waveforms by solving one-dimensional (1-D) Navier–Stokes equations, and as well as non-linearities (Segers et al., 1997). The arterial tree (103 segments) includes the main arteries of the systemic circulation, as well as a detailed network representation of the cerebral circulation and the coronary circulation. The arterial segments of the model are considered as long tapered tubes, and their compliance is defined by a nonlinear function of pressure and location (Langewouters et al., 1984). The arterial wall behavior is considered to be nonlinear and viscoelastic (Holenstein et al., 1980). The governing equations of the model are acquired *via* integration of the longitudinal momentum and continuity of the Navier–Stokes equations over the arterial cross-section. Given proper boundary conditions, flow and pressure waves at all locations of the systemic arterial tree are obtained by solving the governing equations using an implicit finite-difference scheme. Local arterial compliance is computed by approximating PWV as an inverse power function of arterial lumen diameter (Reymond et al., 2009). The resistance of the distal vasculature is considered by coupling the terminal vessels with three-element Windkessel models. At the proximal end, the arterial tree either receives a prescribed input aortic flow waveform or is coupled with a time-varying elastance model for the contractility of the left ventricle (Suga and Sagawa, 1974; Sagawa et al., 1977). In the present work, the simulations used the elastance model for the contractility of the left ventricle as a proximal boundary condition. More details on the 1-D model can be found in the original publications (Reymond et al., 2009, 2011). This model has been thoroughly validated against *in vivo* data and was found to be able to provide accurate pressure and flow waves, particularly with respect to shape and wave details.

**Figure 1:**
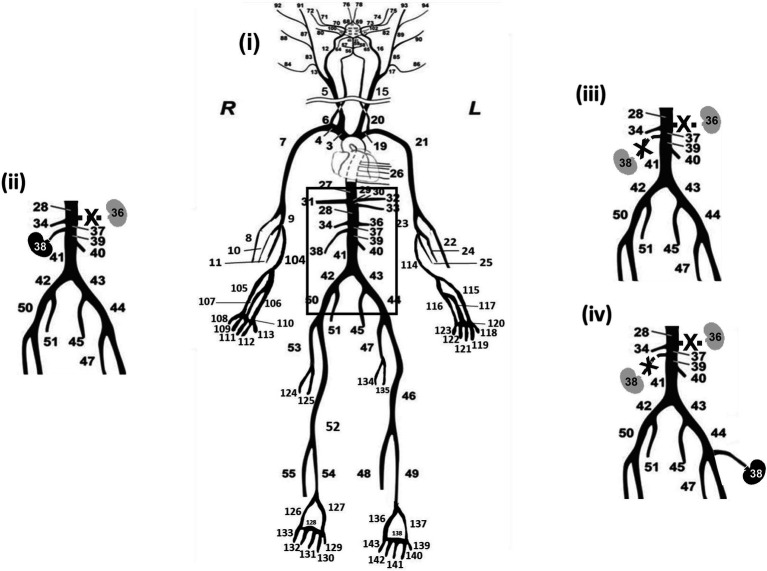
Representation of the complete arterial topology (used in the computer model with the hand and foot topologies, adapted from Reymond et al., 2009). **(i)** Control with two kidneys configuration (2KDN), kidneys in their usual anatomical location on the aorta. **(ii)** One kidney configuration (1KDN), the left renal artery (segment 36) is disconnected from the model. **(iii)** Zero kidneys configuration (0KDN), simulated by removing both the right and the left renal arteries, segments 36 and 38. **(iv)** Transplanted configuration (TX), both renal arteries were disconnected, the right renal artery (segment 38) was attached into the external iliac artery (segment 44).

The arterial topology of the initial model, as described above, was extended by Obeid et al. (2017) to account for additional distal arterial segments including hand and foot topologies (radial and tibial arteries), combined and terminated with three-element Windkessel models. The extended version consists of 143 segments (Figure 1i). The dimensions and properties of the extended arterial tree parts were taken from the literature and completed with data from patient CT scans. The simulated pressure and flow waves in the hand and foot arteries were in good qualitative agreement with the published *in vivo* measurements (Obeid et al., 2017).

### Estimation of Pulse Wave Velocity

**Figure 2:**
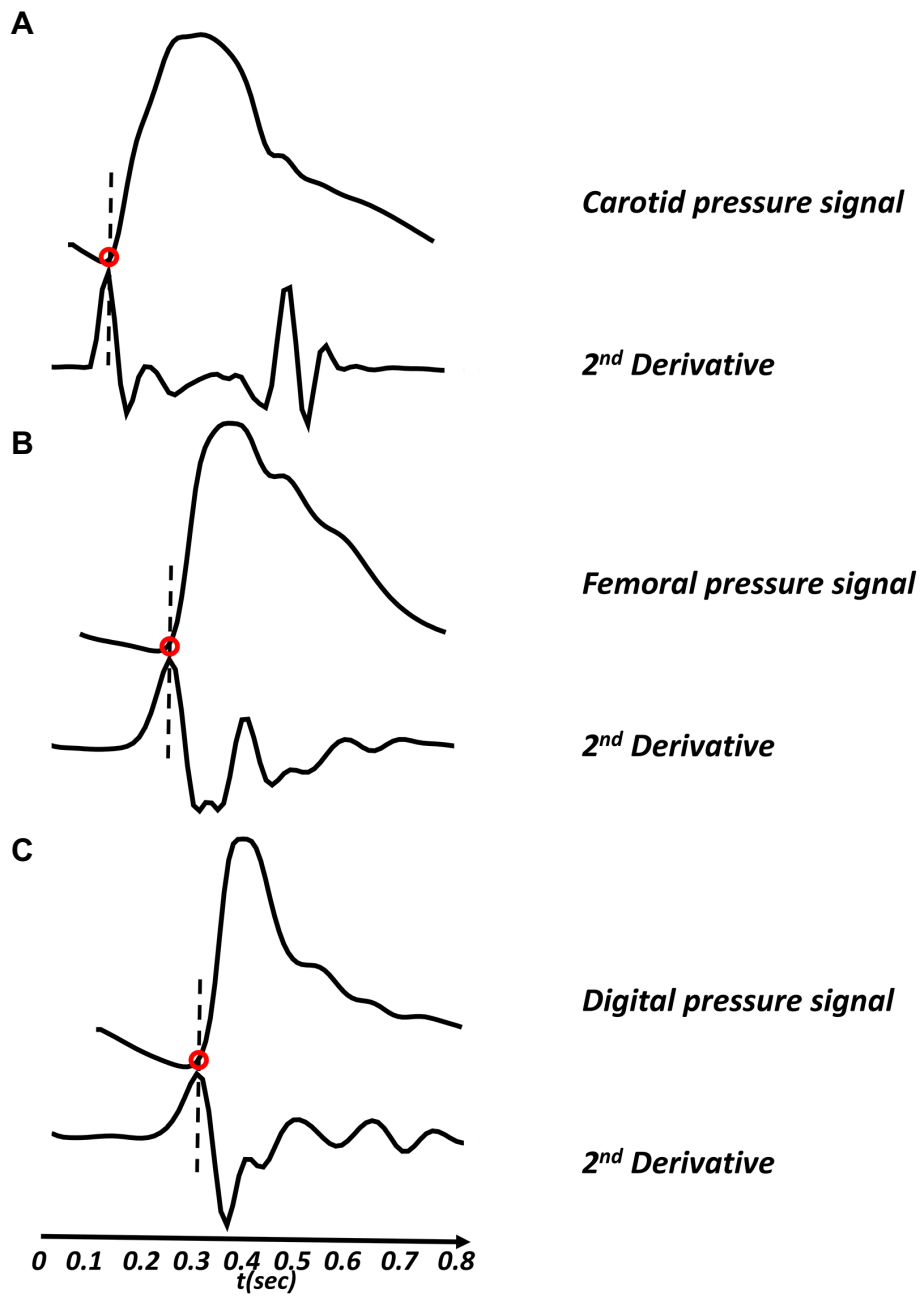
Pulse transit time (PTT) calculation. The figure shows application of Matlab-based second derivative algorithm using the foot-to-foot method. **(A)** Carotid pressure signal (top) with its second derivative (bottom), **(B)** femoral pressure signal (top) with its second derivative (bottom), and **(C)** digital pressure signal (top) with its second derivative (bottom).

**Figure 3:**
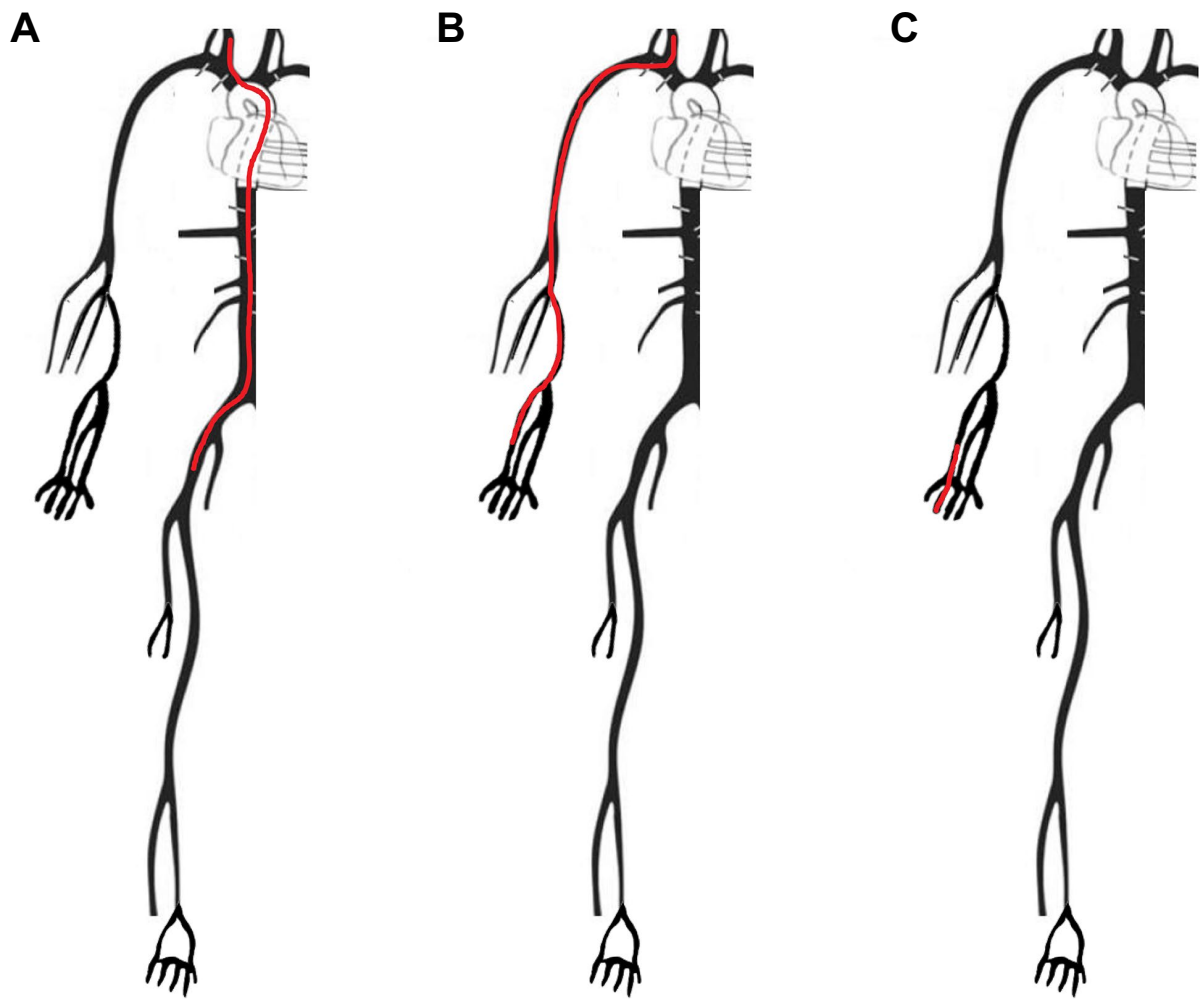
Arterial paths as measured in the different methods of the model, by adding up the lengths of the different segments. **(A)** Carotid-femoral path (Δx_cf) used for the calculation of the cfPWV, measured from the common carotid to the femoral artery *via* the external iliac. **(B)** Carotid-radial path (Δx_cr) used for the crPWV calculation, measured from the common carotid to the radial artery *via* the brachial artery. **(C)** Radial-digital path (Δx_rd) used for the rdPWV calculation, measured from the radial to the palmar digital artery.

The cfPWV, crPWV, and rdPWV were determined by measuring the foot-to-foot PTT between the pressure signals at the carotid and the femoral arteries (cfPTT), the carotid and the radial arteries (crPTT), and the radial and proper palmar digital arteries (rdPTT). The foot-to-foot algorithm using the maximum second derivative was implemented in Matlab (Mathworks, Natick, Massachusetts, United States). The method uses the time point where the maximum second derivative occurs to calculate the foot of the pressure wave, Figure 2. In order to ensure that the short-time delays between radial and finger arteries are captured, the sampling frequency for all the BP waveforms was set to 900 Hz, an order of magnitude higher than 100 Hz which is the threshold value for temporal resolution suggested by Gaddum et al. (2013). The specific value was selected as a fair trade-off between computational time and high signal fidelity. The carotid-femoral distance was determined as the distance from the common carotid artery to the femoral artery (Figure 3A), resulting in Δx_cf = 66 cm. Given that the precise length of every arterial segment of the 1-D model was known, the distance between two arterial sites was computed by summing the individual arterial lengths of all segments within the carotid-femoral path. The carotid-radial (Figure 3B) and the radial-digital distances (Figure 3C) were determined by the summation of the lengths of the arterial segments that connected the two corresponding nodes and were found to be equal to Δx_cr = 70 cm and Δx_rd = 19 cm, respectively. Finally, the value of PWV was calculated by dividing the travel distance by the PTT.

### Numerical Simulation of Staged Kidney Removal

The previously validated human arterial tree model was adapted in order to mimic different stages of kidney disease. Alterations in the arterial tree were performed in terms of geometry, while all the systemic and heart model parameters of the *in silico* model were kept unchanged and equal to their default values (Reymond et al., 2009). Specifically, four model configurations were simulated and are illustrated in Figure 1:

- Two kidneys (2KDN, control): the right and left renal arteries (terminal segments 38 and 36) were located in their usual anatomical location on the aorta in the 1-D model.
- One kidney (1KDN): to simulate a patient with one functional kidney or a kidney-donor subject, we created a configuration characterized by removal of the left renal artery (terminal segment 36) from the systemic circulation, as might be done during organ donation surgery (Friedewald and Ho, 2020).
- No kidneys (0KDN): to simulate the arterial configuration in end-stage renal disease (ESRD), both the right and the left renal arteries (terminal segments 38 and 36) were removed. Indeed, in end-stage kidney disease, the damaged kidney can no longer filter blood effectively. This results in a cascade of physiological signals which leads to shunting of most of the usual 15%–20% of cardiac output directed at the kidneys toward the rest of the systemic circulation (Johnson et al., 2014).
- Kidney transplantation (TX): to simulate a renal transplant recipient, we removed both renal arteries, as to generate the above-mentioned 0KDN state of disease, and then attached a right renal artery (terminal segment 38) into the external iliac artery (terminal segment 44) of the transplant recipient (Karusseit, 2018). The terminal three-element Windkessel was omitted from the external iliac and a branch was added bearing the vascular properties of the right renal artery.

Subsequently, we calculated cfPWV, crPWV, and rdPWV for all four different configurations. Cardiovascular parameters of the default-model configuration are shown in Table 1, representing a healthy subject (2KDN) with *in silico* distensibility values.

**Table 1:** Cardiovascular parameters of the default-model configuration (2KDN).

Parameter	2KDN (*In silico*)
Aortic SBP (mmHg)	118
Aortic _early-_DBP (mmHg)	91
Aortic _end-_DBP (mmHg)	73
Mean arterial pressure (mmHg)	88
Radial SBP (mmHg)	129
Digital _early-_DBP (mmHg)	101
Digital _end-_DBP (mmHg)	73
Heart rate (bpm)	75
Cardiac output (L/min)	5
Aortic distensibility (KPa^−1^ × 10^−3^)	27
Aortic PWV_Bramwell-Hill_ (m/s)	5.9

*In silico* distensibility values. SBP, systolic blood pressure; DBP, diastolic blood pressure. PWV_Bramwell-Hill_ = (*ρ* × distensibility)^−1/2^; *ρ*, blood density 1,050 kg/m^3^.

### Variations of Arterial Distensibility

After simulating the impact of removing one or more kidneys with constant model parameters, we performed a set of simulations, first using the model’s default value of distensibility for each arterial segment and then forcing changes from −20%, −40%, −80% to 20%, 40%, 80% of its default value. The changes in arterial distensibility were identical for every segment of the arterial network. By changing the distensibility simultaneously in each segment of the arterial tree, we were able to assess their influence on arterial PWV. A description on how segment distensibility and compliance were determined has been provided by Reymond et al. (2009). Finally, since, biologically aorta stiffens to a greater degree than peripheral arteries, we used an empirical approach to simulate non-uniform changes in distensibility by reducing distensibility by 40% (for diameter >14 mm), 25% (for diameter more than 11 to ≤14 mm), 15% (more than 8 to ≤11 mm), 10% (more than 3 to ≤8 mm), and 5% (for diameter ≤3 mm). We then used the non-uniform increase in stiffness with 0KDN and compared the results to the default distensibility data with 2KDN.

### *In vivo* and Interpolated Distensibility

In addition to the above-mentioned simulations, we used *in vivo* distensibility data taken from the literature on ESRD. The average age of the *in vivo* ESRD population was 56–63 years. For these simulations, the aortic, carotid, brachial, and radial arteries were altered using *in vivo* values, as shown in Table 2 (ESRD distensibility values). In addition to these known segmental distensibility values, we interpolated the *in vivo* data to estimate distensibility for the remaining arterial segments with a 30% decrease of the default *in silico* value to mimic diseased segments. The interpolated segments were the femoral, tibial, coronary, cerebral, digital, and dorsal as shown in Table 2. The default-model distensibility values (*in silico*) for all arterial segments were shown also for the sake of comparison between healthy and diseased cases.

**Table 2:** Distensibility of default values (*in silico*) versus distensibility of end-stage renal disease (ESRD; *in vivo*).

Arterial segments	Distensibility (KPa^−1^ × 10^−3^)		PWV_Bramwell-Hill_ (*m/s*)	
	*2KDN* *In silico*	*0KDN* *In vivo*	*2KDN* *In silico*	*0KDN* *In vivo*
Aorta	27.0	21.0	5.9	6.7
Carotid	23.0	18.0	6.4	7.3
Femoral	10.6	6.6	9.5	12.0
Tibial	9.0	5.0	10.3	13.8
Brachial	7.3	3.5	11.4	16.5
Radial	6.4	2.6	12.2	19.1
Coronary	6.1	4.5	12.5	14.5
Cerebral	8.1	4.1	10.8	15.2
Digital	5.3	3.4	13.4	16.7
Dorsal	5.7	3.2	12.9	17.2

*In vivo* data references: aorta (Briet et al., 2006); carotid (Zanoli et al., 2019); brachial (Verbeke et al., 2007); and radial (Mourad et al., 1997). PWV_Bramwell-Hill_ (*m/s*), pulse wave velocity calculated using the Bramwell–Hill equation.

The aortic and carotid distensibility values were extracted from Briet et al. (2006), Edwards et al. (2008), Zanoli et al. (2019) while the radial and brachial values were obtained from Mourad et al. (1997), Verbeke et al. (2007), respectively. Using Bramwell–Hill equation (Bramwell and Hill, 1922), PWVs were reported for all arterial segments as shown in Table 2.

## Results

### Blood Pressure and Kidney Configurations With Default Distensibility

**Figure 4:**
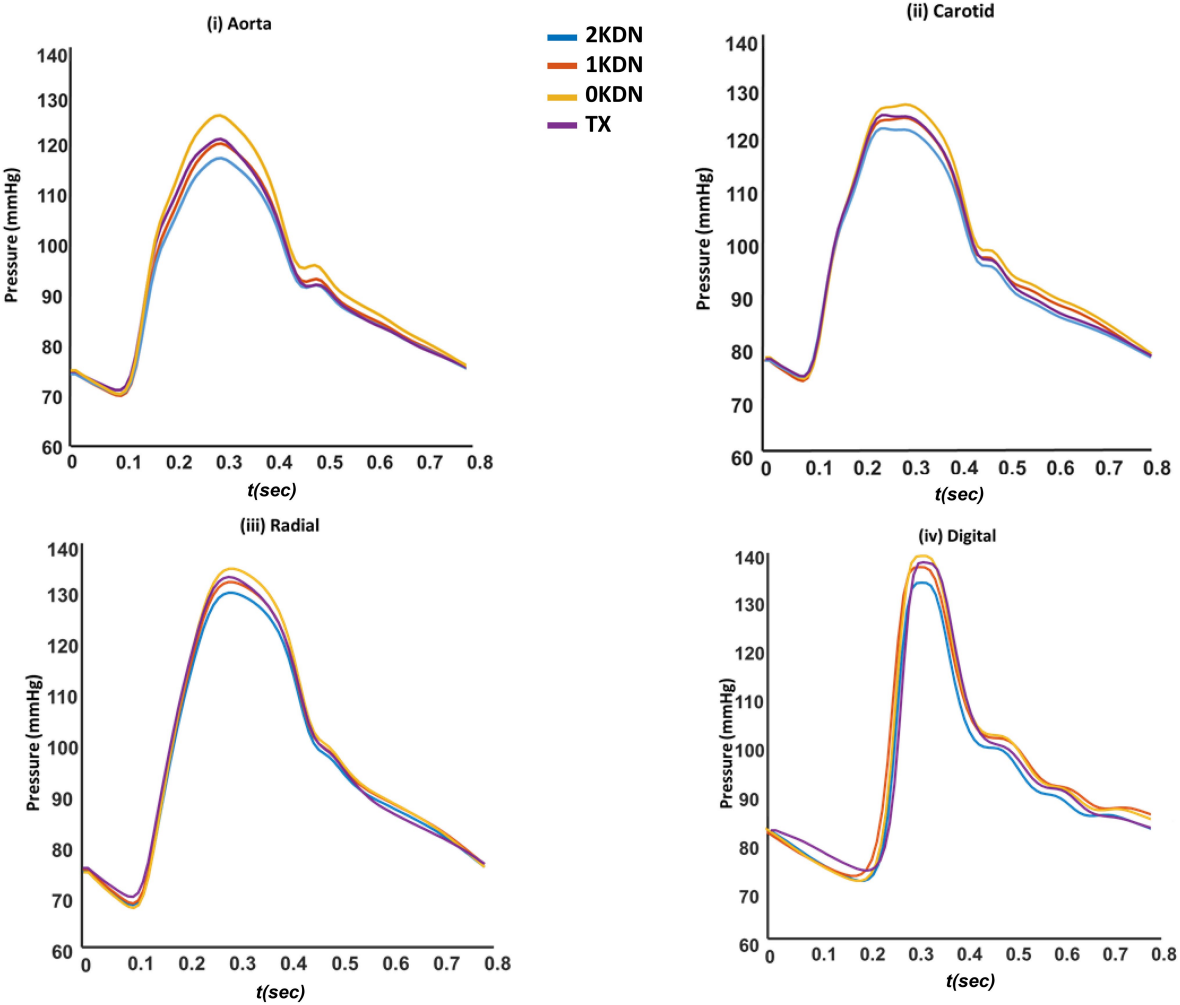
Variations of **(i)** aortic, **(ii)** carotid, **(iii)** radial, and **(iv)** digital blood pressure waveforms in the four kidney configurations (default distensibility and *in silico*).

**Figure 5:**
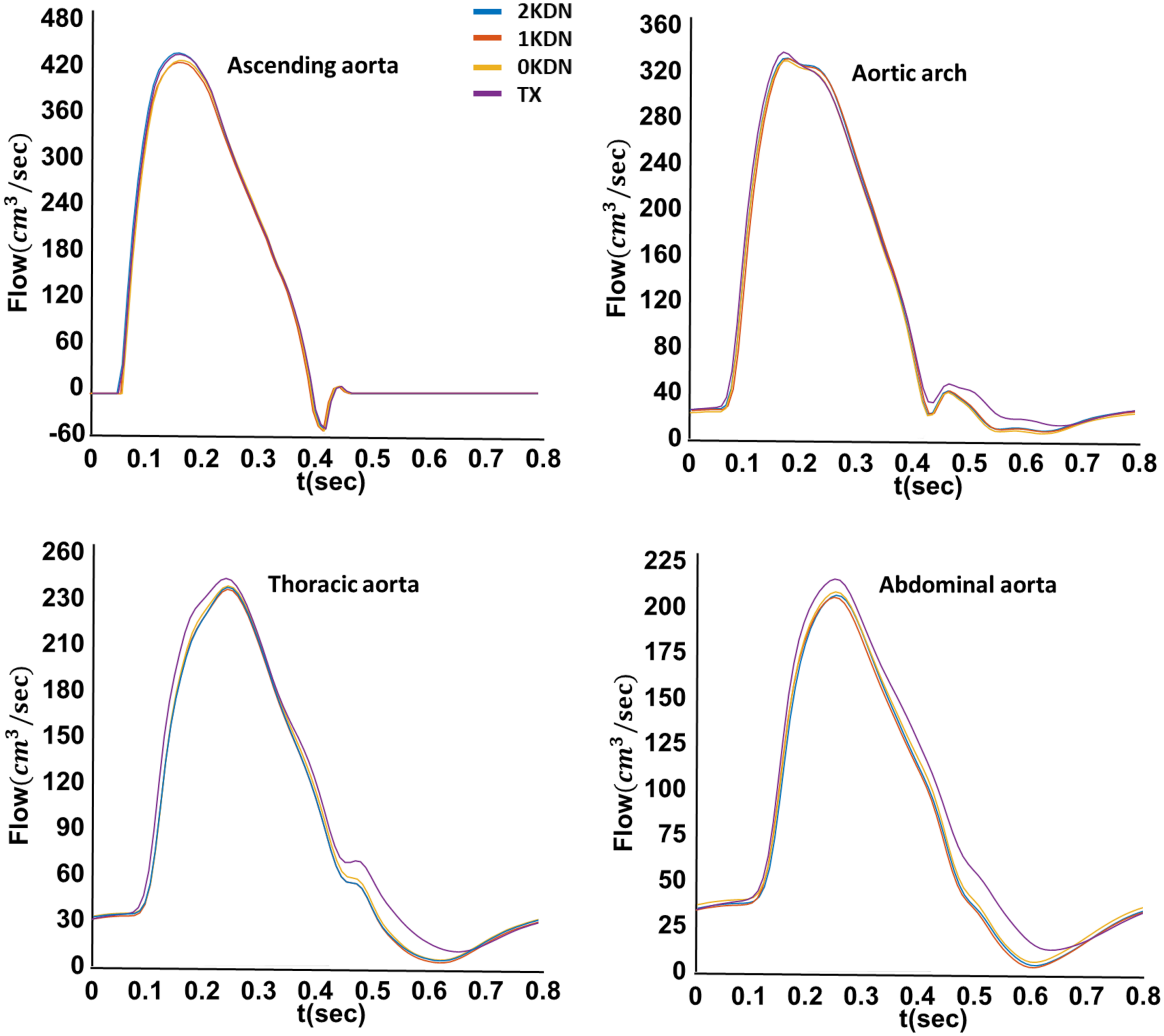
Variations of the aortic flow waveforms in the four simulated configurations at four different locations in the aorta (default distensibility and *in silico*).

**Figure 6:**
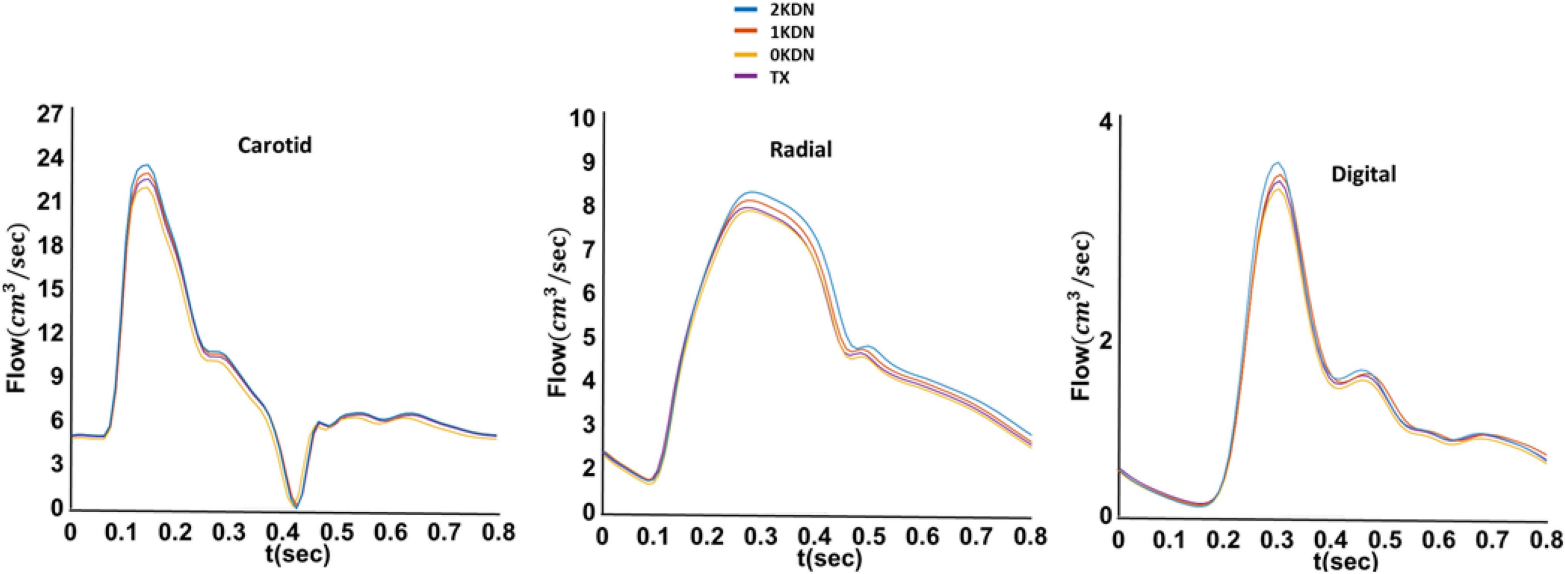
Variations of the carotid, radial, and digital flow waveforms in the four simulated configurations (default distensibility and *in silico*).

Systolic blood pressure (SBP) was increased at all arterial sites with the stepwise removal of the kidneys. Similarly, early diastolic blood pressure (_early-_DBP) was increased, but to a lesser degree, while the end-diastolic BP (_end-_DBP) remained relatively stable. The concomitant changes in SBP without significant changes in _end-_DBP led to an increase in PP along the arterial tree. Importantly, without any kidneys, aortic and carotid SBP increased by 9 mmHg and 7 mmHg compared to the control kidney configuration. Similar changes occurred to the radial SBP (+6 mmHg) and digital SBP (+7 mmHg). Additionally, a restoration of the BP values was observed in the TX configuration similar to 1KDN. Detailed presentation of the variations in the pressure values for all renal configurations is reported in Table 3. In addition, the respective variations in the shape of the BP waves are illustrated in Figure 4. Variations in the aortic shape of flow waves are illustrated in Figure 5 with the four kidney configurations at different locations including ascending aorta, aortic arch, thoracic aorta, and abdominal aorta (below the renal arteries). The flow rate decreased significantly from the ascending aorta (430 cm^3^/s) to abdominal aorta (215 cm^3^/s). The aortic flow waves look similar for the ascending aorta and the aortic arch in the four configurations. However, the changes become more observable in the thoracic and abdominal aorta, areas which are closer to the renal arteries. Interestingly, the flow wave in abdominal aorta (below the native renal arteries) was higher in TX configuration compared to other configurations (2KDN, 1KDN, and 0KDN). Moreover, with the stepwise removal of the kidneys, there was a stepwise decrease in peak flow in the carotid, radial, and digital arteries, but with the TX configuration, the flow patterns became similar to 1KDN configuration. Figure 6 shows the flow waves in the carotid, radial, and digital arteries where the flow rate decreased significantly from the carotid (24 cm^3^/s) to the digital artery (4 cm^3^/s).

**Table 3:** Changes in blood pressure with different kidney configurations using *in silico* distensibility data.

Parameter (mmHg)	2KDN	1KDN	0KDN	TX
Aortic SBP	118	122	127	123
Aortic _early-_DBP	91	92	98	91
Aortic _end-_DBP	73	72	74	75
Aortic PP	45	50	53	48
Carotid SBP	122	125	129	126
Carotid _early-_DBP	97	98	100	97
Carotid _end-_DBP	76	74	75	77
Carotid PP	46	51	54	49
Radial SBP	129	132	135	133
Radial _early-_DBP	98	99	100	99
Radial _end-_DBP	70	71	69	72
Radial PP	59	61	66	61
Digital SBP	133	138	141	135
Digital _early-_DBP	101	102	103	102
Digital _end-_DBP	73	75	74	76
Digital PP	60	63	67	59

SBP, systolic blood pressure; DBP, diastolic blood pressure; and PP, pulse pressure.

### Variation of PWV for *in vivo* and *in silico* Distensibility

**Figure 7:**
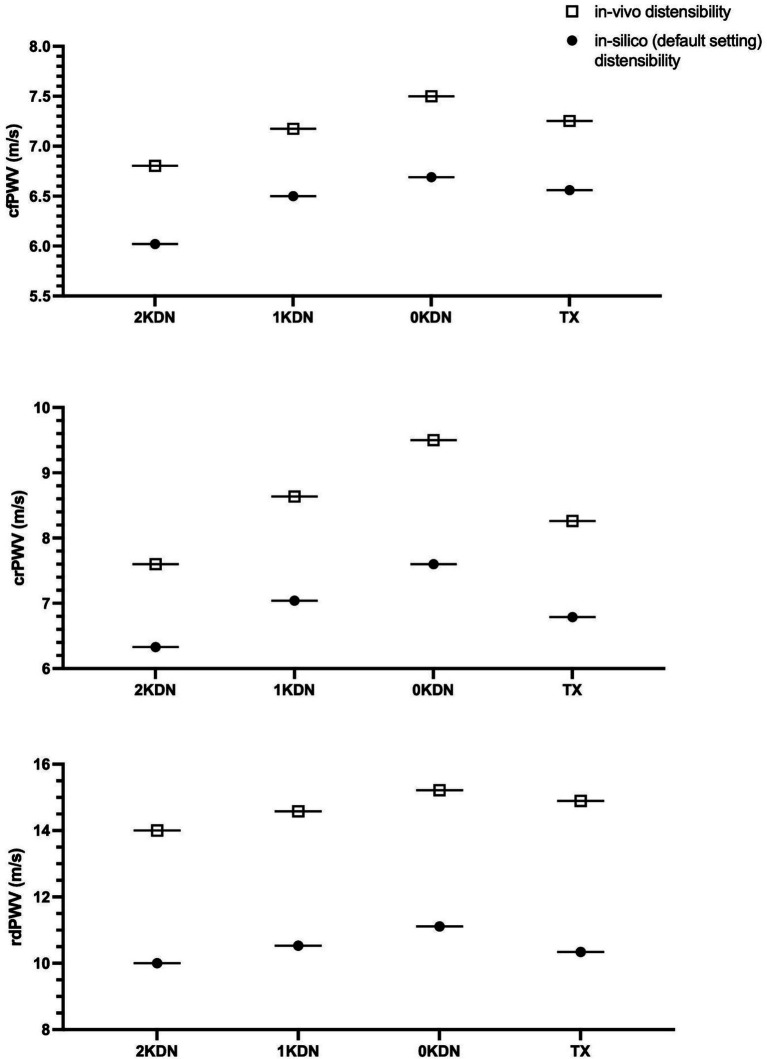
Pulse wave velocity (PWV) results obtained with the literature *in vivo* distensibility data (0KDN) and the model default distensibility data (*in silico*).

The variations of the PWV of each arterial segment under the four configurations using *in vivo* and *in silico* distensibility values are illustrated in Figure 7. The PWV values varied with removal or addition of kidneys using both *in vivo* and *in silico* data. In particular, an increase was observed in all *in silico* PWVs with increasing severity from 2KDN to 1KDN, and to 0KDN, namely, 6.02, 6.50, and 6.69 m/s for cfPWV, 6.33, 7.04, and 7.6 m/s for crPWV, and 10, 10.53, and 11.11 m/s for rdPWV, respectively. Similar variations were observed with *in vivo* PWVs with higher PWV values. The relative difference in stiffness from control to 0KDN was higher in the case of crPWV (15%) in comparison with the increase observed for cfPWV (11%). When the right renal artery was transplanted to the left external iliac artery (TX configuration), we observed a restoration of the PWVs to values close to those reported for the 1KDN configuration, i.e., for *in silico* PWVs, 6.56, 6.79, and 10.34 m/s, for cfPWV, crPWV, and rdPWVs, respectively.

### Variation of PWV for Different Degrees of Arterial Distensibility

**Figure 8:**
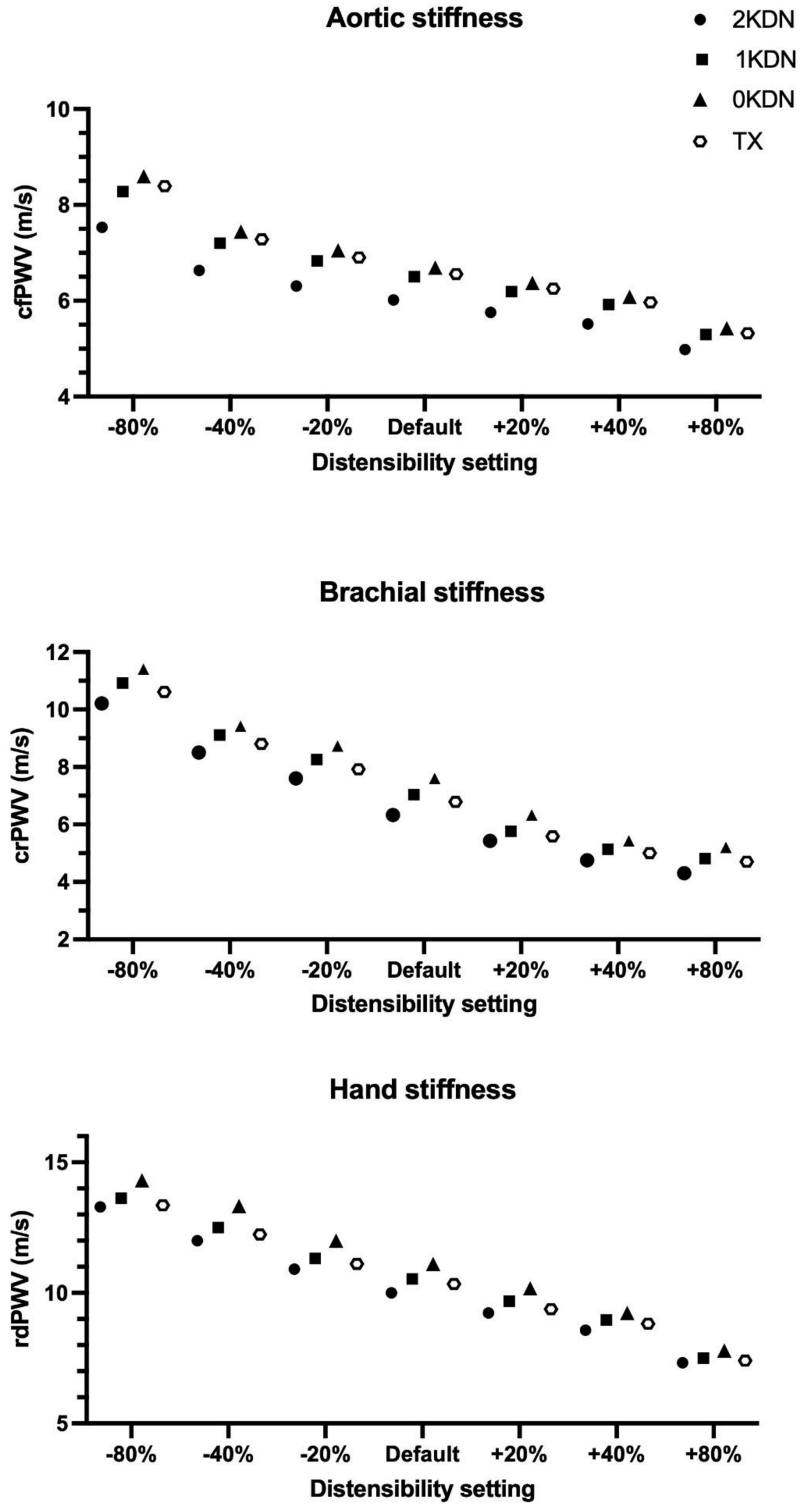
Regional arterial stiffness (AS) with varying degrees of distensibility per renal disease status simulation (*in silico* distensibility).

**Figure 9:**
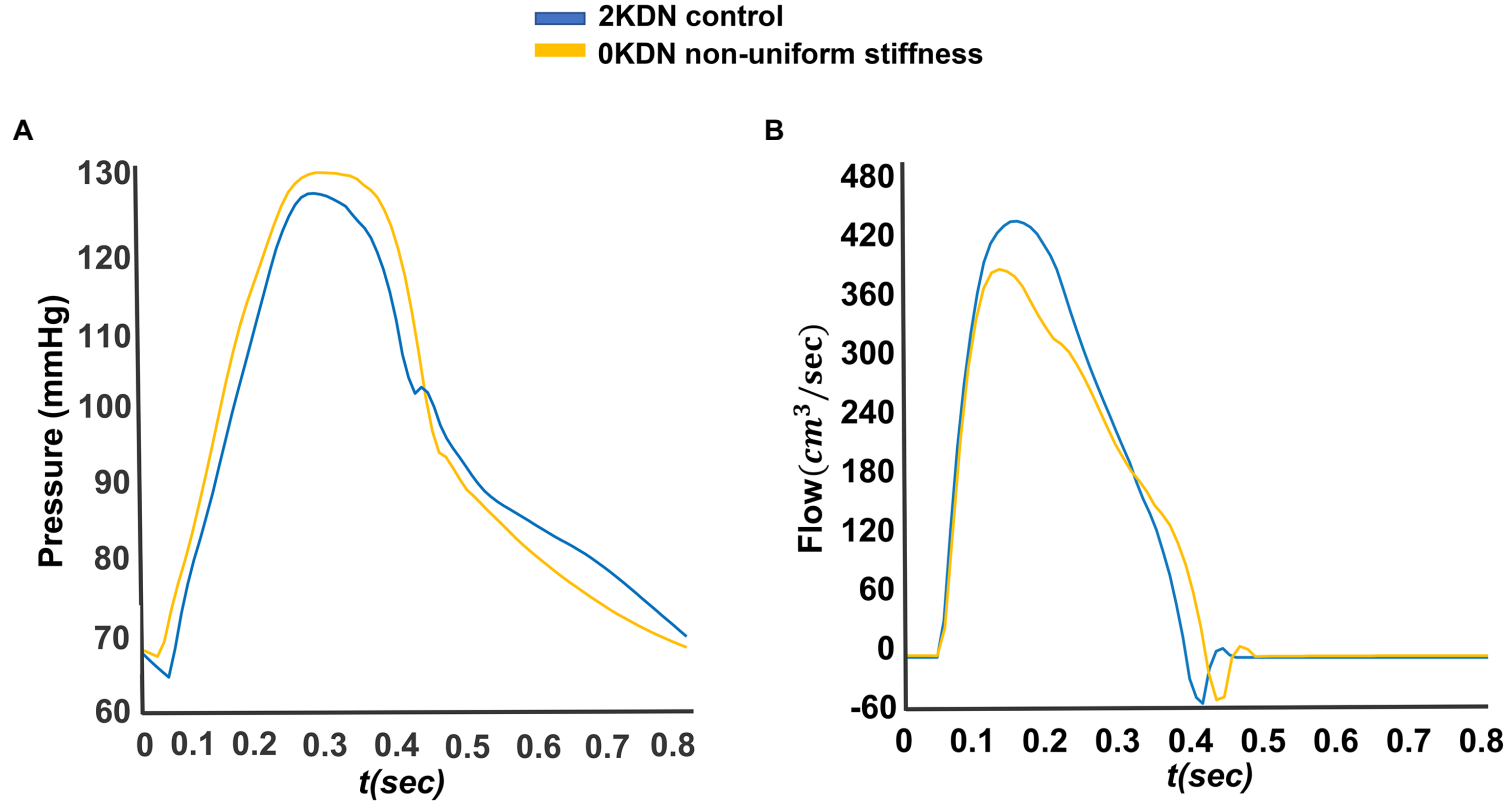
Variations of the aortic pressure and flow waveforms. **(A)** Pressure waveforms in the non-uniform stiffness configuration without kidneys (0KDN) compared to the default distensibility with 2KDN (control). **(B)** Flow waveforms in the non-uniform stiffness configuration without kidneys (0KDN) compared to the default distensibility with 2KDN (control).

Moreover, notable variations were observed when varying degrees of arterial distensibility were inputted into the configurations. The variations of the three PWV values with the staged removal of the kidneys under different distensibility values are reported in Figure 8. Given that aortic stiffening is more profound than stiffening in peripheral arteries, the non-uniform increase in stiffness without kidneys (0KDN) was compared to the values of PWV using default distensibility with 2KDN (control). As expected cfPWV, crPWV, and rdPWV increased by 20% (7.25 m/s), 40% (8.86 m/s), and 19% (11.94 m/s), respectively. Figure 9 shows the differences in the aortic pressure and flow wave patterns between these two configurations. Indeed, the peak and the duration of peak systolic pressure increased in 0KDN with non-uniform increase in distensibility (Figure 9A). However, the peak aortic flow was reduced, but it was compensated by a higher duration of ejection and a late systolic increase in flow (Figure 9B).

## Discussion

In this numerical model study, we used a 1-D model to simulate hemodynamic effects, and their impact on functional AS, using configurations that would reduce renal blood flow, as occurs with a clinical reduction in glomerular filtration rate. The observation that *in silico* central and peripheral PWV values change with varying stages of renal disease is in accordance with available *in vivo* data, and even more importantly, our findings suggest that the PWV variations observed in the clinical configuration of various stages of kidney disease may partially be attributed to biomechanical alterations of the arterial tree, and in turn BP.

The kidneys are high-flow and low-resistance organs, which receive 15%–20% of the cardiac output. With progressive kidney disease, an important reduction in renal blood flow occurs; a clinical situation which may be simplified as a state in which the renal arteries are functionally absent. This state is believed to significantly alter the biomechanical properties of the cardiovascular system which could subsequentially modify AS. To examine this hypothesis, we used a validated human arterial tree model and ran numerical simulations to determine PWV in various configurations that mimic different stages of kidney disease. Besides the loss of renal function from kidney injury, there are clinical settings in which one of the kidneys is removed surgically, either for kidney donation purposes or for the treatment of kidney cancer. Following a kidney donation, which is generally performed on younger, healthy individuals, there seems to be a medium to long-term increased risk of hypertension and cardiovascular disease (Boudville et al., 2006; Garg et al., 2008; Mjoen et al., 2011, 2014). However, in the short term, ambulatory BP studies show that BP remains stable when measured 6 months after kidney donation (Prasad et al., 2008). After kidney donation, the glomerular filtration rate declines by only 30% despite a reduction of nephron mass of 50%. These observations suggest that renal hyperfiltration and other biological compensatory mechanisms maintain BP within the normal range, at least in the short term. While the BP response to nephrectomy for cancer treatment is less well studied, radical nephrectomy is associated with increased risk of hypertension and cardiovascular disease mortality, as compared to partial nephrectomy in some studies (Shah et al., 2019; Capitanio et al., 2020). Nonetheless, the interplay between increased AS and alterations in the arterial network in the presence of reduced renal blood flow has not been specifically addressed. Therefore, the use of a simulation model provides us with the unique opportunity to isolate the effects of kidney as a vascular organ.

Our study shows an increase in SBP with staged removal of the kidneys. *In vivo* data from the literature has shown that elevated SBP is associated with the development and progression of renal dysfunction (Young et al., 2002; Bell et al., 2012). This positive association between SBP and the risk of ESRD might be mediated through the augmentation of PP with increasing SBP. Indeed, aortic stiffness results in the transmission of high pulsatile energy (PP) into the microcirculation of target organs such as kidneys. This pulsatile energy can damage the organ’s microcirculation and result in tissue injury. For this reason, PP has been frequently used as surrogate of AS, when a rise in PP is associated with reduced arterial compliance (Niiranen et al., 2019). In agreement with *in vivo* observations, the results from this numerical simulation study show an increase in PP along the arterial tree along the arterial tree with the staged removal of the kidneys. This is also important given that current evidence suggests that PP may be more sensitive to renal disease outcomes compared to SBP and DBP (Arulkumaran et al., 2010; Geng et al., 2019).

Our study also shows an increase in AS with staged removal of the kidneys. Arterial remodeling and early vascular aging occur from the early stages of renal disease and amplify with its progression (Kimoto et al., 2003; Briet et al., 2006; Ferreira et al., 2017). Ferreira et al. have previously demonstrated that aortic PWV is significantly higher in patients with kidney failure in comparison with both normotensive and hypertensive controls, in individuals below 60 years of age. Their findings suggest that PWV has prognostic value *vis-à-vis* future structural arterial alterations, as well as a role to play in guiding therapeutic interventions (Ferreira et al., 2017). Furthermore, it has been observed that AS in ESRD patients is enhanced independently of age and BP (London et al., 1996). Consequently, aortic PWV itself has been established as an index of cardiovascular risk and mortality in this population (Guerin et al., 2001; London, 2018). The results from this paper’s numerical simulations showed that removing both kidneys increased cfPWV and crPWV by 11% and 15%, respectively. This might be explained by the nonlinear pressure-area relationship, which leads to an increased PWV in peripheral arterial. Yet, the observed increase in SBP was higher in the aorta than in the peripheral arteries. This evidence leads us to believe that in the 1KDN and 0KDN states, an additional *area-dependent effect* on stiffening might be taking place. More specifically, an increase in BP leads to an overall increase in stiffness (pressure-dependent effect) in both central and peripheral arteries. Moreover, considering the pressure-area dependency, the larger (central) arteries will present a smaller increase in stiffness, whereas the smaller (peripheral) arteries will present a larger increase in stiffness (area-dependent effect). The two effects will act cumulatively and the total increase in the crPWV will be higher than in the cfPWV. Given that PWV was computed using the foot-to-foot method and thus, using the diastolic points of the pulse wave, increase in PWV values in this study may not be explained by the small changes in the _end_-DBP.

In ESRD, it has been shown that cfPWV increases overtime but that crPWV decreases with time, suggesting that changes in PWV ratio (a measure of AS gradient) over time were not only guided by an increase in cfPWV but also by a reduction in crPWV (Utescu et al., 2013; Laucyte-Cibulskiene et al., 2019). Investigators have shown that PWV ratio can be a better prognostic marker than cfPWV (Fortier et al., 2015; Bao et al., 2019). How and why crPWV are reduced over time in ESRD remains a matter to be investigated. Using the proposed model, we have first assumed no changes in the characteristics of various vascular segments and have used various configurations with more or less proportional changes in the aorta versus peripheral arteries. To the best of our capacity, we have extrapolated vascular wall properties from the known values that were available, but only a limited number of vascular segments were available for ESRD patients. Finally, using a non-uniform approach to increased stiffness from aorta to the peripheral segments, cfPWV increased by 20%, whereas crPWV increased by 40%. As such, the PWV ratio did not follow the same pattern as with *in vivo* findings previously published (Utescu et al., 2013; Fortier et al., 2015; Bao et al., 2019; Laucyte-Cibulskiene et al., 2019). This discrepancy underlines the importance of other possible factors that affect vascular remodeling over a longer period of time and are likely to involve outward remodeling and intrinsic changes in the vascular wall composition and vascular tone.

The flow patterns in the proximal aorta did not change significantly in the various configurations of this study. However, in the configuration where both kidneys were removed and one kidney was attached to the external iliac artery, we noticed a significant increase in the abdominal aortic blood flow below the original sites of the renal arteries. Using the non-uniform increase in the stiffness of arterial tree and removing the kidneys, peak systolic blood flow was reduced, but this was compensated by a higher late systolic flow rate and an increase ejection duration. While detailed *in vivo* flow patterns in various stages of kidney disease are lacking, this model provides a novel insight on potential patterns of blood flow in ESRD.

The approach used has some limitations that need to be recognized. First, the staged removal of kidneys was created solely considering anatomical changes in the arterial tree. Indeed, kidneys have multiple functions and play a major role in the regulation of BP through various mechanisms involving neurohormonal adaptation through natriuretic peptides, pressure natriuresis, and downregulation of sympathetic tone, vasopressin release, and the renin-angiotensin-aldosterone system (Wadei and Textor, 2012). Furthermore, kidneys contribute significantly in the regulation of calcium phosphate homeostasis, which is deregulated with CKD and this deregulation is intimately involved in the process of vascular calcification (Reiss et al., 2018). Moreover, we recognize that there is a chronic adaptation of the cardiovascular system (heart and vascular wall) in response to reduction in renal blood flow and reduced renal function. In advanced kidney disease, uremic toxins, endothelial dysfunction, anemia, vascular calcification, and chronic microinflammation all lead to cardiovascular changes. In addition, cardiovascular biology and function may also be modified by cardiovascular drugs, dialysis treatment, and the arteriovenous fistulas in some patients. It is therefore difficult to isolate a specific process related to CKD-mediated changes in the vascular wall. Investigators tend to forget that besides these effects, kidneys are high-flow and low-resistance organs and that a gradual decrease in kidney function is associated with reduced renal blood flow and increased renal vascular resistance. As such, the originality of our approach was in quantifying the impact of the kidney, as a vascular organ, rather than a metabolic organ, using the 1-D numerical simulation model to dissociate this component from the biological component of the kidney function. Second, the distensibility values taken from the literature were only available for some arterial sites (Table 2). In order to address and minimize the impact of said limitation, we chose to perform interpolation in order to approximate missing distensibility values for the major arteries. The average age of the *in vivo* literature population was around 58 years however, whereas the *in silico* default-model distensibility values correspond to a 30-year-old healthy subject. However in this study, we used both *in vivo* and *in silico* PWVs. While this does not correspond to actual measurements, it constitutes a highly representative approximation of human data. Finally, our model did not incorporate changes in the cardiac parameter. However, we know that generally, in advanced kidney disease, cardiac output may increase due to anemia (Metivier et al., 2000).

## Conclusion

In conclusion, using a human 1-D arterial tree model, we have demonstrated that the increases in BP and regional AS, which are observed in clinical studies, are at least in part due to the purely biomechanical effects of staged reduction in renal blood flow. Observed changes in central and peripheral PWV values with varying stages of renal function were in line with previously reported clinical data. Further analyses are needed to mimic more realistic configurations for the ESRD patients considering potential variations in cardiac output and the mean arterial pressure.

## Data Availability Statement

The raw data supporting the conclusions of this article will be made available by the authors, without undue reservation.

## Disclosure

All authors have reported that they have no relationships relevant to the contents of this paper to disclose.

## Author Contributions

HO and MA conceived of the presented idea. HO and VB developed the theory and performed the computations. CF and PS verified the analytical methods. PS supervised the methods section. MP contributed to the figures production and data analysis. NS and MA encouraged HO and VB to investigate and supervised the findings of this work. All authors contributed to the article and approved the submitted version.

## Conflict of Interest

The authors declare that the research was conducted in the absence of any commercial or financial relationships that could be construed as a potential conflict of interest.

## Publisher’s Note

All claims expressed in this article are solely those of the authors and do not necessarily represent those of their affiliated organizations, or those of the publisher, the editors and the reviewers. Any product that may be evaluated in this article, or claim that may be made by its manufacturer, is not guaranteed or endorsed by the publisher.
